# Nanoparticles based on retinoic acid caped with ferrocenium: a novel synthesized targetable nanoparticle both with anti-cancer effect and drug loading capacity†

**DOI:** 10.1039/c9ra02472g

**Published:** 2019-05-23

**Authors:** Yibo Wang, Bin Zhao, Lu Wang, Wenhuan Bu, Shuwei Liu, Bin Sun

**Affiliations:** Department of Oral and Maxilloficial Surgery, School and Hospital of Stomatology, Jilin University Changchun 130041 People’s Republic of China sunbin06@sohu.com; Jilin Provincial Key Laboratory of Tooth Development and Bone Remodeling, Jilin University Changchun 130041 People’s Republic of China; Department of Periodontosis, School and Hospital of Stomatology, Jilin University Changchun 130041 People’s Republic of China; State Key Laboratory of Supramolecular Structure and Materials, College of Chemistry, Jilin University Changchun 130012 People’s Republic of China

## Abstract

To date, there is an urgent need for cancer treatment to improve in many ways in order to successfully cure all cancers. Retinoic acid (RA) is a promising anti-cancer drug through influencing cancer stem cells (CSCs). Taxol is a chemotherapy drug for many cancers. To increase the anti-cancer effects of RA and taxol, we created a novel RA nanoparticle, FCRAN, which has the ability of carrying a second anti-cancer drug, taxol, using nanotechnological methods. The results of this study demonstrated that this RA nanoparticle was water-soluble and retained the same effects as RA on cancer cells, such as inhibiting the proliferation of CSCs, inducing the differentiation of CSCs, and enhancing the sensitivity of CSCs to chemotherapeutic drugs. In addition, this RA nanoparticle can be used to carry a second anticancer drug, taxol, to become FCRAN/T and synergistically enhance the anti-cancer effects of both drugs *in vivo*. Interestingly, the FCRAN/T is a targetable anti-cancer nanoparticle in the presence of higher levels of glutathione (GSH) in cancer cells. Our results demonstrate that our novel synthesized nanoparticles not only retain the RA functions, but can also carry a second anticancer drug to play a synergistic anticancer role with good water solubility, in particular FCRAN/T can target cancer cells. Therefore, our novel synthesized targetable anti-cancer nanoparticles have a better application prospect than that of RA or taxol alone.

## Abstract 2

A retinoic acid nanoparticle with the ability of carrying a second anti-cancer drug, taxol, was developed. The anti-cancer nanoparticles were shown to have a better application prospect than that of RA or taxol alone.

## Graphical abstract

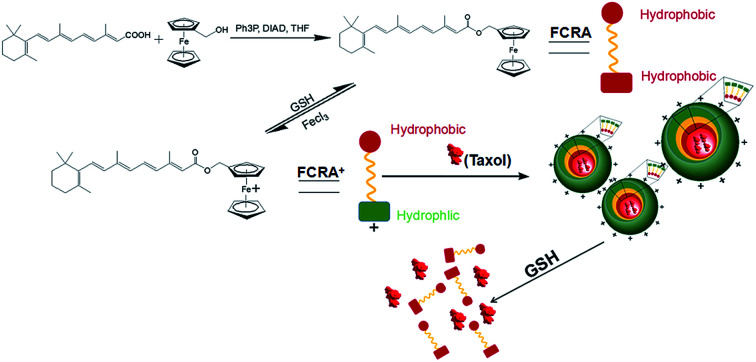

## 1. Introduction

Cancer is a disease with the highest morbidity rate in the world even though scientists have been trying to find efficient treatments for many years. To date, chemotherapy is still the most important treatment for many cancers.^1,2^ At least 50 different types of chemotherapy drugs are currently available to efficiently treat ∼200 different types of cancers. The outcome of chemotherapy, however, is strictly hampered because of drug resistance and the cytotoxicity of healthy cells.^3,4^

Cancer stem cells (CSCs) play an important role in tumorigenesis, drug-resistance and the relapse of cancers.^5–8^ CSCs are a small number of undifferentiated or poorly differentiated cancer cells, and have the characteristics of self-renewal. The higher expression of intracellular and extracellular drug transport systems, and the strong ability of CSCs to proliferate and repair damage to DNA as well as anti-apoptosis make them more tolerant to chemotherapeutic drugs than other differentiated cancer cells, namely non-CSCs.^9,10^ Therefore, CSCs and the other cancer cells, non-CSCs, can have different responses for the same chemotherapy, therefore, different strategies have to be used to fight CSCs and non-CSCs.

Retinoic acid (RA) is the active derivative of vitamin A, a fat-soluble vitamin, which plays key roles in cell growth and differentiation.^11^ RA can induce undifferentiated or poorly differentiated CSCs into differentiated cancer cells, reduce the proliferation and migration ability of CSCs, and increase the sensitivity of CSCs to chemotherapeutic drugs.^12,13^ Previous studies demonstrated that RA can suppress lung, prostate, breast, ovarian, bladder, oral and skin cancers although RA alone is a weak anticancer drug.^14^ RA, however, is insoluble in water, and can cause cytotoxicity to normal cells/tissues at higher dose.^15^

We hypothesized that a nanoparticle may be used as a special carrier to overcome the poor solubility of RA in water, decrease its cytotoxicity and increase its anti-cancer effects or to provide very useful properties, such as stability, specificity, encapsulation, unique size and biocompatibility, to carry RA and others at the same time. In our previous study we demonstrated that a ferrocenium capped RA is not only water soluble and has stronger anti-cancer effects compared with RA alone, but it also inhibits the proliferation and differentiation of CSCs.^16^ In the current study, RA was used directly to make a nano-carrier, which retained RA anti-activity, had nanoparticle properties, and could also carry another anti-cancer drug, taxol, to enhance its anti-cancer effects.

## 2. Experimental section

### 2.1 Syntheses of ferrocenylmethylretinoate (FCRA) and oxidized FCRA (FCRA^+^)

Ferrocenylmethylretinoate (FCRA) and oxidized FCRA (FCRA^+^) were prepared according to the methods described in our previous publication.^16^ Briefly, 648.18 mg of ferrocenyl methanol (3 mmol), 1.18 g of PPh3 (ferrocenemethano, 4.5 mmol, SIGMA-ALORIC H, ST. Louis. MO, USA), 901.2 mg of ATRA (all trans retinoic acid, 3 mmol, SIGMA-ALORICH) were added to 20 mL of THF followed by stirring until all the reagents were fully dissolved. Then, 0.8 g of DIAD (diisopropyl azodicarboxylate, 4.5 mmol, SIGMA-ALORICH) was added under a N_2_ atmosphere at 0 °C, followed by continuous stirring for 2 h at room temperature, and concentration using a rotary evaporator at 30 °C to obtain a viscous oil-like liquid, which was then purified by column chromatography using ethylacetate/petroleum ether (v/v = 2/8) to obtain ferrocenyl ratinoic acid, FCRA. To produce FCRA^+^, 4.98 mg of FCRA (0.1 mmol) was dissolved in 2 mL of CH_2_Cl_2_, and mixed with a solution of 27.3 mg of FeCl_3_·6H_2_O (0.1 mmol) dissolved in 2 mL of CH_3_CN. Following oxidization by FeCl_3_, the solvent was evaporated *in vacuo* to obtain green solid FCRA^+^.

### 2.2 Preparation and characterization of FCRA^+^ nanoparticles (FCRAN)

The green solid FCRA^+^, prepared as detailed above, was dissolved in 1 mL of anhydrous ethanol, then 9 mL of ddH_2_O was added whilst simultaneously shaking the solution ultrasonically for 30 min. The solution was kept at room temperature overnight. The next day, this solution was dialyzed using a dialysis bag (molecular weight cutoff 3500) in ddH_2_O until the solution was no longer green, followed by filtration using a 0.22 μm sterile filter to obtain brown yellow FCRAN (1 μmol mL^−1^). The morphology of FCRAN with or without GSH was observed using an FEI-Tecnai-F20 high resolution transmission electron microscope (TEM, FEI Company, Hillsboro, USA). The size of the nanoparticles and the potential of the nano ions were measured by dynamic light scattering (DSL, Malvin instruments, UK).

### 2.3 Taxol loading and release by FCRAN

To enhance the anti-cancer effects, herein FCRAN carried a second chemotherapy medication, taxol which is a mitotic inhibitor. To enable taxol loading and retain the properties of FCRAN, first a loading assay was performed, as follows. Taxol is a water insoluble medication. When taxol and FCRAN were mixed, un-encapsulated taxol settled down after high speed centrifugation. Taxol and FCRAN mixed at different ratios of FCRAN to taxol were used (mol_FCRAN_/mol_taxol_), 0 : 1, 1 : 1, 2 : 1, 4 : 1 and 6 : 1, samples were mixed at room temperature at 5000 rpm. The supernatant liquid was discarded, the pellets were dried at 50 °C and then dissolved in ethanol, followed by measurement by UV-visible spectrophotometry (Shimadzu, Japan). The maximum taxol loading efficiency by FCRAN was calculated using the following equation:Max loading efficiency (%) = (mol_taxol_/mol_FCRAN_) × 100

Next, a release assay was performed. 20 mL of an aqueous solution of taxol-loaded FCRAN (1 μmol mL^−1^) was prepared and divided into groups A and B (10 mL each). GSH was added to group A (final concentration of 10 mmol L^−1^, simulated intracellular concentration of tumor^17^), and ddH_2_O was added to group B. At time points of 0, 5, 15, 30 and 60 minutes. The precipitate was separated by centrifugation (5000 rpm). The ultraviolet absorption peak at 230 nm (UV absorption peak wavelength of taxol) was measured by UV-visible spectrophotometry using a Shimadzu 3100 instrument. The drug release rate of taxol (%) = (original solution absorption value − measured supernatant absorption value)/original solution absorption value × 100%.

### 2.4 Preparation and characterization of FCRAN/taxol nanoparticles (FCRAN/T)

FCRA^+^ was dissolved in 1 mL of anhydrous ethanol containing 1 μmol of taxol, then 9 mL of ddH_2_O was added whilst simultaneously shaking the solution ultrasonically for 30 min. The solution was kept at room temperature overnight. The next day, this solution was dialyzed using a dialysis bag (molecular weight cutoff 3500) in ddH_2_O until the solution was no longer green, followed by filtration using a 0.22 μm sterile filter to obtain brown yellow FCRAN/T (1 μm mL^−1^). The morphology of FCRAN/T with or without GSH was observed using an FEI-Tecnai-F20 high resolution transmission electron microscope. The size of the nanoparticles and the potential of the nano ions were measured by dynamic light scattering. Infrared spectroscopy (Nicolet Instrument Corporation, USA) was used to detect if FCRAN and taxol were assembled together.

### 2.5 Cell culture

KB (a human squamous carcinoma cell line, from ATCC), CAL-27 (a human tongue squamous carcinoma cell line, from ATCC), A2870 (a human ovarian cancer cell line, from ATCC), and HSG (a human submandibular gland cell line, from ATCC) were cultured in an H-DMEM medium (Gibco Company, USA) containing 10% fetal bovine serum (Gibco Company, USA), 100 U mL^−1^ penicillin and 100 g mL^−1^ streptomycin (Gibco Company, USA). Cells were incubated at 37 °C in humidified 5% CO_2_. The medium was replaced every 3 days.

### 2.6 Sorting the cancer stem cells

Hoechst 33342 dual wavelength fluorescence analysis is a means of purifying and characterizing adult tissue stem cells, and Hoechst unstained cells were described as the side population (SP) cells, namely, stem cells.^18,19^ KB, CAL-27 and A2870 cells were harvested, warmed at 37 °C for 10 min, stained with 5 μg mL^−1^ of Hoechst 33342 in the medium at 37 °C for 90 min, then sorted at 420–470 nm (blue) and 660–680 nm (red) using a FACSDiva (Becton Dickinson, San Jose, CA, USA). The cell line with the highest population of SP cells or CSCs, A2870 cells was used for further experiments. Verapamil (100 μmol L^−1^, SIGMA-ALORICH, USA), an inhibitor of Hoechst 33342 efflux was used to confirm that Hoechst 33342 staining was specific.

### 2.7 Colony formation assay

CSCs which had been sorted from A2870 cells were cultured at 3 × 10^2^ cells/30 mm plate with 20 μM of FCRAN or 20 μM of RA alone for 5 days. The control group contained only culture medium, the medium was then changed and continued to be cultured for 2 weeks. Cells were washed with PBS and fixed with 4% paraformaldehyde for 10 min and stained with 0.5% crystal violet for 5 min. The colonies were counted, and the colony formation rates were calculated using the following formula: colony formation rate = (number of colony/inoculated cells at the beginning) × 100%.

### 2.8 Cell migration assay

CSCs which had been sorted from A2870 cells were cultured at 2 × 10^5^ cells per well in 6-well plates with 20 μM of FCRAN or 20 μM of all-trans retinoic acid for 3 days. The control group contained only culture medium. Cells from each group were harvested with 0.25% trypsin, spun and suspended with serum-free H-DMEM medium. Cells from each group were transferred into the upper compartment at 3 × 10^4^ cells per well in a 8 μm Transwell plate. 500 μL of H-DMEM medium containing 10% fetal bovine serum was added into the lower compartment, and cultured for 24 hours. Then, the cells were fixed with 4% paraformaldehyde for 10 min. The Transwells were gently wiped off with a cotton swab and stained with 0.5% of crystal violet for 5 min. Cells on the Transwell were observed, photographed and counted undera microscope to evaluate whether the cells had migrated to the polycarbonate microporous membrane. Five fields were counted from each group.

### 2.9 Drug resistance assay

CSCs which had been sorted from A2870 cells were cultured at 3 × 10^3^ cells per well in 96 well plates with all-trans retinoic acid (Ra), taxol (Ta), all-trans retinoic acid and taxol (Ra + Ta), FCRAN/T (Ft) for 24 hours. The control group contained only culture medium. The final concentration of FCRAN/Tand all-trans retinoic acid were 10 and 20 μM, respectively. Because the molar ratio of FCRAN to Ta in FCRAN/T was 10 : 1, the amount of Ta in the Ta group and the Ra + Ta group was the same as that in the Ft group, which was 1/10 of Ft concentration. Then, 20 μL of 5 mg mL^−1^ working concentration of MTT solution was added to every cell well. The cells were further incubated for 4 h. Finally, 150 μL of DMSO was added to each well to replace the medium in each well and to dissolve the formazan crystals, and then the absorbance was measured at a test wavelength of 490 nm. The cell viability was calculated using the following formula: percentage cell viability = (absorbance of the experiment samples/absorbance of the control) × 100%.

### 2.10 Gene expression profiles of Oct4 and Sox2

CSCs which had been sorted from A2870 cells were cultured at 2 × 10^5^ cells per well in 6 well plates with 20 μM of FCRAN or 20 μM of all-trans retinoic acid for 2 days. Total RNA was extracted from each group using an miScript SYBR Green PCR Kit (TaKaRa, Japan). Real-time PCR (QPCR) was performed using Oct4 forward primer (5′-GCTGGATGT CAGGGCTCTTTG-3′)/Oct4 reverse primer (5′-TTCAAGAGATTTA TCGA GCACCTTC-3′), and Sox2 forward primer (5′-GTGAGCGCCCTGCA GTACAA-3′)/Sox2 reverse primer (5′-GCGAGTAGGACATGCTGTAGGT G-3′), internal control using β-actin forward primer (5′-TGGCA CCCAGCACAATGAA-3′)/β-actin reverse primer (5′-CTAAGTCATAGTCC GCCTAGAAGCA-3′).

### 2.11 Assay of targeted anti-cancer effect of FCRAN/T *in vitro*

KB and HSG cells were cultured at 3 × 10^3^ cells per well in 96-well plates for 24 h. Each cell was divided into two groups and treated with FCRAN/T and Ta for 24 h respectively. The final concentration of FCRAN/T was 0, 1.25, 2.5, 5, 10 and 20 μM. Because the molar ratio of FCRAN to Ta in FCRAN/T was 10 : 1, the amount of Ta in the Ta group was the same as that in the FCRAN/T group, which was 1/10 of FCRAN/T concentration. Then, 20 μL of 5 mg mL^−1^ working concentration of MTT solution was added to every cell well. The cells were further incubated for 4 h. Finally, 150 μL of DMSO was added to each well to replace the medium in each well and to dissolve the formazan crystals, and then the absorbance was measured at a test wavelength of 490 nm. The cell viability was calculated using the following formula: percentage cell viability = (absorbance of the experiment samples/absorbance of the control) × 100%.

### 2.12 Experiments of anti-cancer effect of FCRAN/T *in vivo*

This study was performed in strict accordance with the NIH guidelines for the care and use of laboratory animals (NIH Publication no. 85-23 Rev. 1985) and were approved by the Institutional Animal Care and Use Committee of Jilin University (Jilin, China). In this study, male BALB/c-nu mice (18–20 g of body weight, 8 weeks old), obtained from the Chinese Academy of Medical Sciences Institute of Zoology (Peiking, China), were treated with a KB cell line for *in vivo* experiments. On day 0, 2 × 10^6^ of KB cells (200 μL) were subcutaneously injected into the right side groin of the mice. Three days post injection of KB cells, the mice were randomly divided into five groups (5 mice per group), control (saline, 100 μL per mice), RA (60 μM, 100 μL per mice), FCRAN (60 μM, 100 μL per mice), taxol (60 μM, 100 μL per mice), and FCRAN/T (60 μM, 100 μL per mice), and were injected with the corresponding treatment around the injection site of the KB cells. Tumor sizes, length and width were measure on day 14, then measured twice/week till day 25. After measurement on day 25, the tumors were removed and weighed. The tumor volumes were calculated using the following formula: length × width^2^/2.

### 2.13 Statistical analysis

The experimental data were expressed in the form of mean ± standard deviation, and analyzed by one-way ANOVA. When *P* < 0.05, the statistical significance was significant.

## 3. Results and discussion

**Fig. 1:**
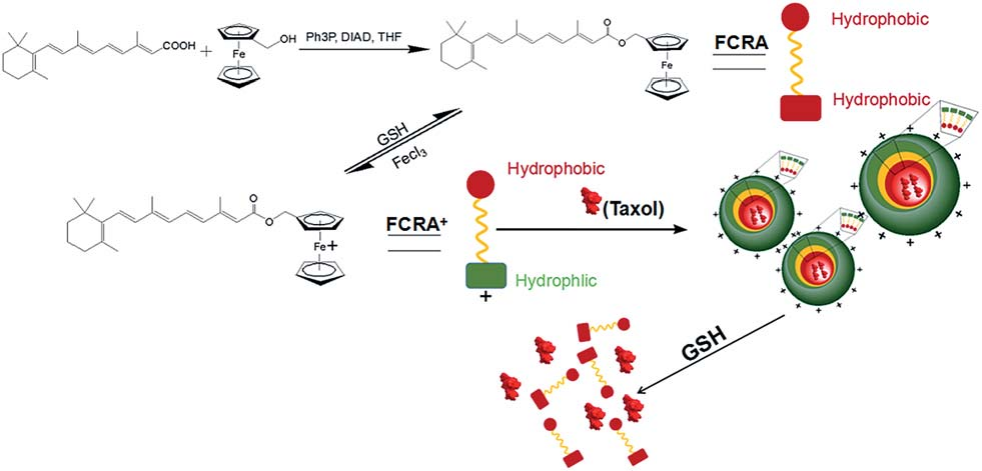
Scheme of FCRAN/T synthesis and synergistic effects.

In recent years, there has been a rapid development in nanotechnology. Many studies have involved the used of nanotechnology to create carriers to efficiently deliver anti-cancer drugs.^20,21^ In our current study, we created a novel nanoparticle which can carry two anti-cancer drugs at the same time and target cancer cells with higher levels of GSH. Fig. 1 is a scheme of the synthesis and synergistic effects of FCRAN/T, which shows that RA was added to a ferrocenyl group through the esterification reaction of retinoic acid and ferrocenylmethanol to obtain FCRA. Further, FCRA was oxidized by FeCl_3_.^16^ The ferrocenium group of FCRA^+^ is positively charged, making FCRA^+^ an amphiphilic molecule and resulting in its self-assembly, in aqueous solution, to a nanoparticle, FCRAN. The inner part of this nanoparticle is hydrophobic thereby enabling it to carry another hydrophobic anticancer drug, such as taxol to become FCRAN/T. The ferrocenium group in FCRAN/T is sensitive to reduced GSH, which results in FCRAN/T losing its polarity to become a hydrophobic molecule, FCRAN/T immediately dissociates, and taxol and ferrocene retinoic acid become free and active forms which are able to synergistically kill targeted cancer cells when FCRAN/T enters cancer cells containing a high level of GSH. It is known that the level of GSH is elevated in many cancer cells while normal cells have lower levels of GSH.^17^

### 3.1 Characteristics of ferrocenyl retinoic acid nanoparticles (FCRAN)

**Fig. 2:**
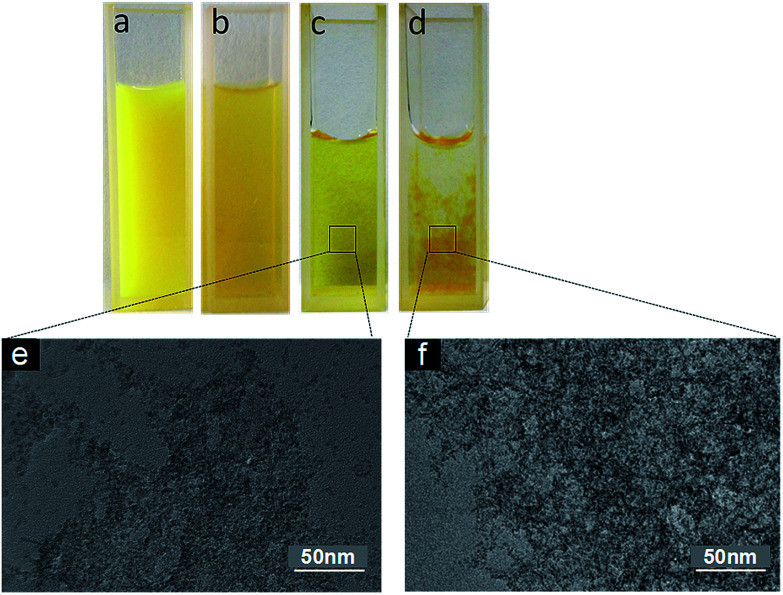
Physical appearance of FCRA and FCRAN. (a) Insoluble FCRA in ddH_2_O before oxidation. (b) Soluble FCRA in ddH_2_O after oxidation. (c) FCRAN in ddH_2_O. (d) FCRAN in ddH_2_O after GSH was added. (e) TEM image of (c). (f) TEM image of (d).

FCRA, which was synthesized herein, is still insoluble in water. It has a bright yellow color if dissolved in DMSO (Fig. 2a). After oxidization with FeCl_3_ and dilution in water, FCRA^+^ immediately forms a turbid liquid with a slightly dark yellow color (Fig. 2b). The water solubility of oxidized FCRA (FCRA^+^) is significantly increased although FCRA^+^ could not be completely dissolved in water because FCRA^+^ is still an amphiphilic molecule rather than a completely hydrophilic molecule (Fig. 2b). The oxidized FCRA^+^, however, can assemble itself to form a completely water-soluble nanoparticle (FCRAN) when FCRA^+^ is first dissolved in a small amount of ethanol, then diluted 10-fold with water, it becomes a slightly dark yellow transparent liquid (Fig. 2c). When reductive glutathione (GSH) is added to the solution, the FCRAN dissociates resulting in precipitation (Fig. 2d).

Transmission electron microscopy (TEM) and dynamic light scattering (DLS) were used to evaluate the morphology and size distribution of FCRAN. From the TEM images it can be seen that FCRAN are round particles of similar size (Fig. 2e). When GSH was added, the FCRAN dissociated into disordered flocs (Fig. 2f).

DLS showed that the diameter of the FCRAN was about 8–20 nm, with an average of 13.76 ± 0.2 nm (Fig. S1a†); the nanoparticles were positively charged and the average Zeta potential was 24.6 ± 0.1 MV (Fig. S1b†).

### 3.2 Characteristics of FCRAN/T

**Fig. 3:**
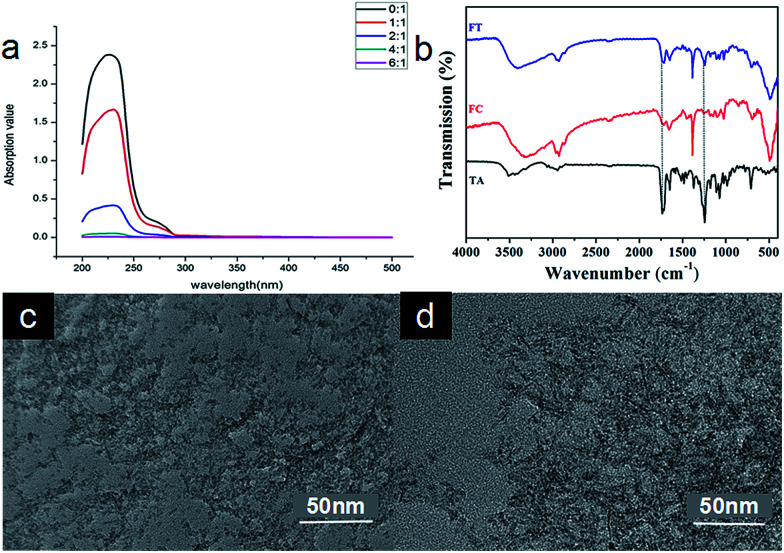
Characteristics of FCRAN/T. (a) UV absorption of residual taxol for different ratios of FCRAN and taxol. (b) FTIR of FCRAN/T (Ft), FCRAN (Fc) and taxol (TA). The infrared spectra of FCRAN/T (Ft) and FCRAN (Fc) were approximately the same, but the peak shape of FCRAN/T (Ft) was similar to that of taxol (TA) at 1250 cm^−1^ and 1750 cm^−1^. This suggests that FCRAN and taxol are assembled nicely at ration of 10 : 1. (c) TEM image of FCRAN/P. (d) TEM image of FCRAN/P with GSH.

The core of FCRAN is hydrophobic, and should carry the hydrophobic anticancer drug, taxol. To assess whether FCRAN can really carry taxol, and the maximum loading amount, a loading assay was performed using different ratios of FCRAN to taxol: 0 : 1, 1 : 1, 2 : 1, 4 : 1 and 6 : 1. The absorption of taxol at 230 nm was measured and from the data obtained it was demonstrated that unloaded or residual taxol gradually decreased as the ratio increased (Fig. 3a). There was no unloaded or residual taxol at a ratio of 6 : 1, which indicates that all the taxol was encapsulated in the FCRAN to form FCRAN/T (Fig. 3a) if the ratio is greater than or equal to 6 : 1, and the maximum taxol loading rate of FCRAN/T is 16.7%. So in all further experiments, we used a ratio of 10 : 1.

To further confirm that FCRAN and taxol were present in FCRAN/T, infrared spectra were measured. Fig. 3b clearly shows that FCRAN/T had all similar peaks from both FCRAN and taxol in the infrared spectra of FCRAN/T. This suggests that FCRAN and taxol are assembled nicely at a ratio of 10 : 1.

Next, the morphology of FCRAN and FCRAN/T was examined by TEM and showed that FCRAN/P was similar to FCRAN with round particle shapes and an even distribution (Fig. 2e and 3c). GSH could dissociate the FCRAN/P resulting in disorder flocculation (Fig. 3d).

The results of DLS showed that the size of FCRAN/P was 13.86 ± 0.1 nm (Fig. S2a†), which was slightly larger than that of FCRAN. FCRAN/still had positive charges, and the average Zeta potential decreased slightly to 22.8 ± 0.3 MV (Fig. S2b†).

Many studies show that GSH tends to have higher level in many kinds of cancers, like breast, ovarian, head and neck and lung cancers.^17^Fig. 3d showed that GSH could dissociate FCRAN/T. Data from the release assay demonstrated that FCRAN/T was stable in the absence of GSH, and there was no paclitaxel release during the test period (Fig. S3†). FCRAN/T, however, dissociated rapidly in the presence of GSH, about 70% of taxol was released within 5 min, and taxol release reached a maximum, of about 90%, at 15 min (Fig. S3†). This indicates that the feature of GSH sensitivity of FCRAN/T leads to the cancer cell specificity of FCRAN/T, which will be of benefit for target therapy using these novel synthesized nanoparticles.

### 3.3 Evaluating cancer stem cells (CSCs) from different cancer cell lines

**Fig. 4:**
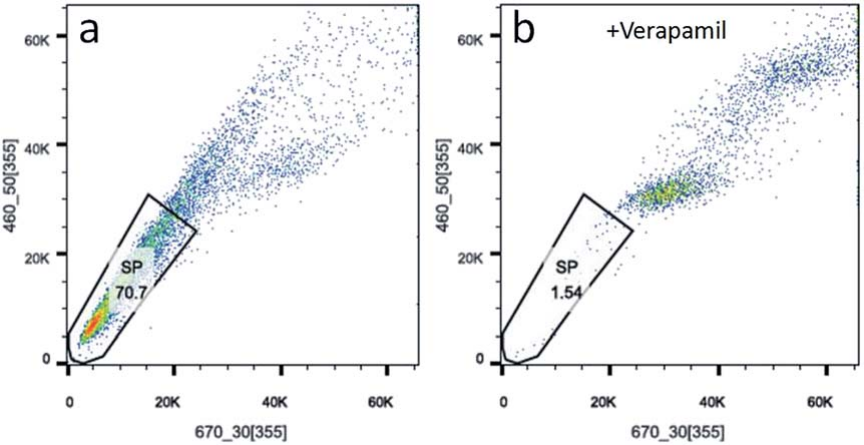
Sorting CSCs from A2870 cells stained with Hoechst 33342. (a) Data from A2870 cells stained with Hoechst 33342. (b) Data from A2870 cells treated with verapamil and stained with Hoechst 33342.

Side population cells (SP cells) can be found to be stem cell-like cells by flow cytometry. Hoechst 33342 dual wavelength fluorescence analysis is a means of enriching CSCs.^22^ In this study, we compared populations of Hoechst 33342 positive SP cells from KB, CAL-27 and A2870 cells. Results from flow cytometry showed that Hoechst 33342 positive was 0.06% for CAL-27 cells, 0.18% for KB cells, and 70.7% for A2870 cells (Fig. 4a and S4†). To further confirm the Hoechst 33342 positive cells were truly positive, verapamil (100 μmol L^−1^), an inhibitor of Hoechst 33342 efflux was used. Data from Fig. 3b clearly demonstrated that CSCs from A2870 cells dramatically decreased. This indicates that A2870 cells have the highest population of CSCs, and can be used to further separate cancer stem cells.

### 3.4 Effects of FCRAN on CSCs of A2870 cells

**Fig. 5:**
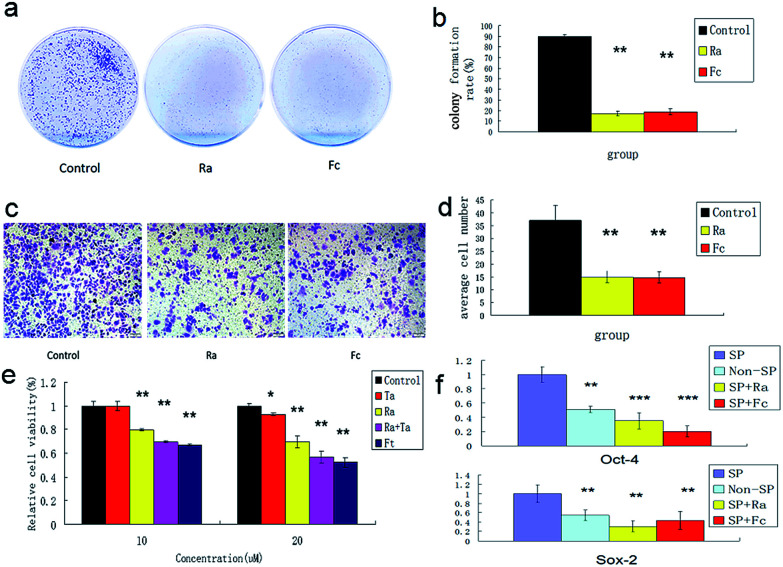
Colony formation and proliferation ability of A2870 cancer stem cells by flat plate colony formation assay. (a) Image data of Colony formation from three groups. (b) Bar graph of data from (a). (c) Image data of CSCs migration of A2870 cells in Transwell plates. (d) Bar graph of data from (c). (e) Effects of paclitaxel on CSCs of A2870 cells. (f) Expression of Sox2 and Oct4 in A 2870 CSCs, non-CSCs, Ra and FCRNA group. Fc stands for FCRAN. Ft stands for FCRAN/T. Ta stands for taxol. Data are shown as means ± SEM.

To understand the effects of FCRAN on CSCs, first, colony formation was used to assess the effect using A2870 cells. CSCs from A2870 cells were sorted by flow cytometry. The results showed that the control group had the highest colony formation with a colony formation rate of 90 ± 2.8%, while FCRAN and RA had dramatically lower and similar colony formation rates of 19 ± 3.1% and 17.3 ± 4.5%, respectively (Fig. 5a and b). The morphologies of the colonies showed that cells from the control group lined up tightly with a smaller round shape while cells from the FCRAN and RA groups were much larger and irregularly shaped (Fig. S5†). Data from migration assay in Transwell plates showed that the highest number of CSCs tried to penetrate polycarbonate micropores in the control group while a significantly lower number of CSCs tried to penetrate polycarbonate micropores in the FCRAN and RA groups (Fig. 5c and d). These results indicate that FCRAN can also inhibit proliferation, colony formation and migration of CSCs, as is the case for RA.

Then, the effect of FCRAN on the sensitivity of CSCs to chemotherapeutic drugs was determined. As shown in Fig. 5e, the cancer stem cells exhibited a marked resistance to taxol (Ta). When Ta acted together with Ra or FCRAN, namely FCRAN/T, the relative cell viability of the cancer stem cells decreased significantly. Ra was similar to Fc in that, there was no obvious difference. This indicates that FCRAN can also increase the sensitivity of cancer stem cells to chemotherapeutic agents, as is the case for RA.

Oct4 and Sox2 mainly express in embryonic stem cells and play an important role in maintaining the pluripotency and self-renewal of embryonic stem cells.^23,24^ Adult cells do not express Oct4 and Sox2, which can be induced into pluripotent stem cells (iPSCs) by transfecting Oct4 and Sox2 genes.^25^ Therefore, the expression levels of these two proteins determines the status of proliferation or differentiation of cancer stem cells and are considered as two markers of stem cells in cancer cells.^26,27^Fig. 5f shows that significantly CSCs from A2870 cells had the highest expressions of Oct4 and Sox2 compared to non-CSCs, FCRAN and RA groups. These results suggest that CSCs from A2870 cells have a higher expression level of Oct4 and Sox2 than non-CSCs, and FCRAN has the same role as RA on the expression of Oct4 and Sox2, which can block further proliferation of CSCs instead of differentiation.

### 3.5 Targeting effect of FCRAN/T on cancer cells

**Fig. 6:**
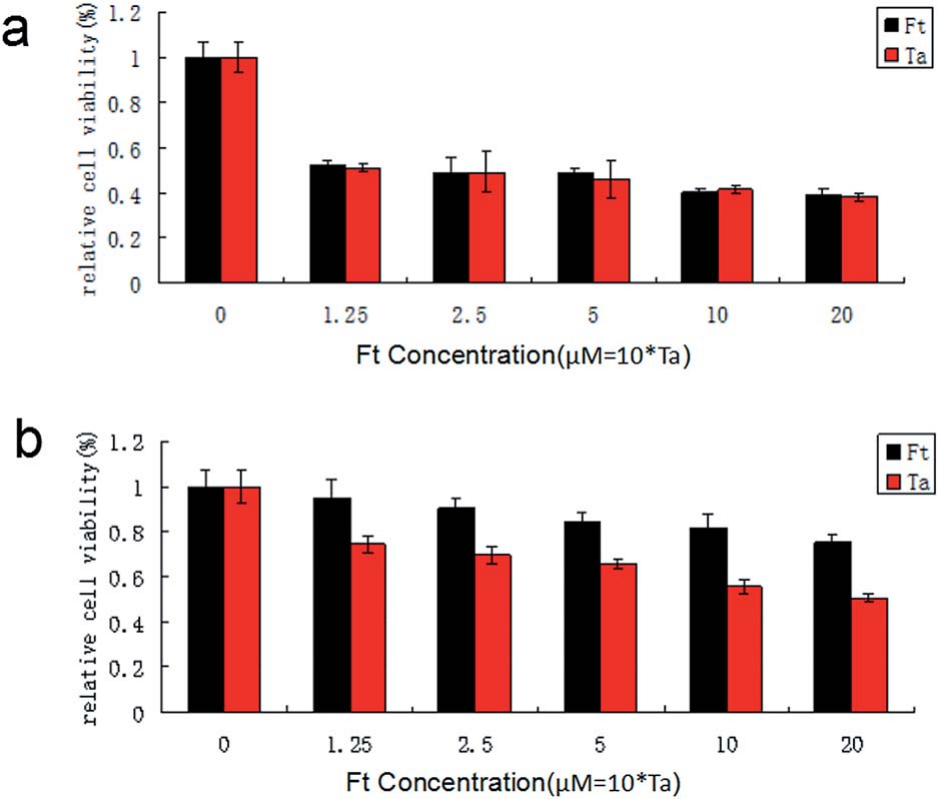
Effect of FCRAN/T (Ft) and taxol (Ta) on cell viability. (a) HSG cells. (b) KB cells. Data are shown as means ± SEM.

Because FCRAN/T are sensitive to GSH, and there is a difference in the GSH content of cancer cells and normal cells, FCRAN/T release taxol rapidly when they enter cancer cells, but do not release or slowly release taxol in normal cells, so they theoretically have a passive cancer cell targeting effect. We examined the cancer cell targeting effects of FCRAN/T on cancer cells and normal cells *in vitro*, used Ta as a positive control. The results showed that FCRAN/T and taxol had a similar killing effect on KB cells (*P* > 0.5), and the relative viability of KB cells decreased to about 50% when the concentration reached 1.25 M (Fig. 6a), but for normal cells (HSG cells), the same concentration of FCRAN/T had less toxicity than taxol (*P* < 0.01). When the concentration reached 20 μM, about 80% of the cells survived (Fig. 6b).

### 3.6 Therapeutic effect of FCRAN/T on cancer *in vivo*

**Fig. 7:**
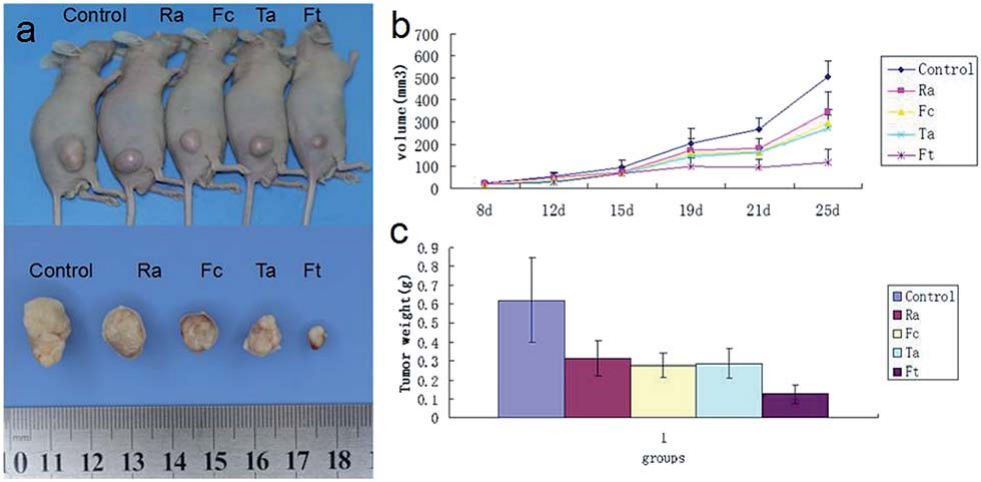
Therapeutic effect of FCRAN/T on a xenogeneic KB tumor model *in vivo*. (a) Representative image of tumor. (b) Curves of tumor size. (c) Bar graph of tumor weight 25 days post-treatment. Fc stands for FCRAN. Ft stands for FCRAN/T. Ta stands for taxol. Data are shown as means ± SEM.

To evaluate the possible clinical application of FCRAN/T, a xenogeneic tumor model of KB cells was used in nude mice. Image data from Fig. 7a clearly showed that the FCRAN/T group had the smallest tumor size of all the groups. Data from the tumor volume and tumor weight further demonstrated detailed differences of each group (Fig. 7b and c), the tumor volume and tumor weight of the FCRAN/T group were the lowest (Fig. 7b and c). These *in vivo* results indicate that FCRAN/T efficiently block KB tumor growth *in vivo*, and the effect of FCRAN/T was significantly higher than that of FCRAN, RA or taxol. Importantly, FCRAN/T indeed has synergistic effects of RA and taxol plus nanoparticle properties and tumor specific target features to GSH.

## 4. Conclusions

CSCs which play an important role in tumorigenesis, and the development, relapse, and chemotherapy resistance of cancers. RA can block the proliferation of CSCs, induce the differentiation of CSCs, and increase the sensitivity of CSCs to chemotherapy drugs. RA alone, however, is insoluble in water, has a weak anti-cancer effect, dose-dependent toxicity, no cancer targeting drug, and can only be used orally or locally in clinics, which limits its clinical application, and efficient treatment. In our current study, we created a novel RA nanoparticle, FCRAN, which had the ability of carrying a second anti-cancer drug, taxol, using nanotechnological methods. The FCRAN/T synthesized herein clearly limits the disadvantages of chemotherapy solely with RA or taxol whilst retaining the anti-cancer effects of RA and taxol. Interestingly, the FCRAN/T achieves a very useful specific effect, targeted cancer cell killing in the GSH condition. *In vivo* data strongly show that FCRAN/T has effective synergistic anti-cancer effects. Therefore, this novel nanoparticle, FCRAN/T has good potential for future clinical application.

## Conflicts of interest

There are no conflicts to declare.

## Supplementary Material

RA-009-C9RA02472G-s001
