# Supplementary material for: Nanoparticles based on retinoic acid caped with ferrocenium: a novel synthesized targetable nanoparticle both with anti-cancer effect and drug loading capacity
Source: RSC Adv. 2019 May 23;9(28):16208–14. doi: 10.1039/c9ra02472g (PMC9064344; doi:10.1039/c9ra02472g)
Supplement: RA-009-C9RA02472G-s001 [file RA-009-C9RA02472G-s001.pdf]

Electronic Supplementary Material (ESI) for RSC Advances.
This journal is © The Royal Society of Chemistry 2019

Nanoparticles based on retinoic acid caped with Ferrocenium: A novel synthesized targetable nanoparticle both with anti-cancer effect and drug loading capacity

Yibo Wang[b],Bin Zhao[b,c], Lu Wang[b], Wenhuan Bu[b], Shuwei Liu[d],Bin Sun[a,b]*

a Department of Oral and maxilloficial surgery, School and Hospital of Stomatology, Jilin University, Changchun, 130041, P. R. China

b Jilin Provincial Key Laboratory of Tooth Development and Bone Remodeling, Jilin University, Changchun, 130041, P. R. China

C Department of periodontosis, School and Hospital of Stomatology, Jilin University, Changchun, 130041, P. R. China

d State Key Laboratory of Supramolecular Structure and Materials, College of Chemistry, Jilin University, Changchun, 130012, P. R. China

* Corresponding author:

Dr. Bin Sun

Department of Oral and maxillofícial surgery , School and Hospital of Stomatology

Jilin University

Changchun, 130041, P. R. China

Email: sunbin06@sohu.com

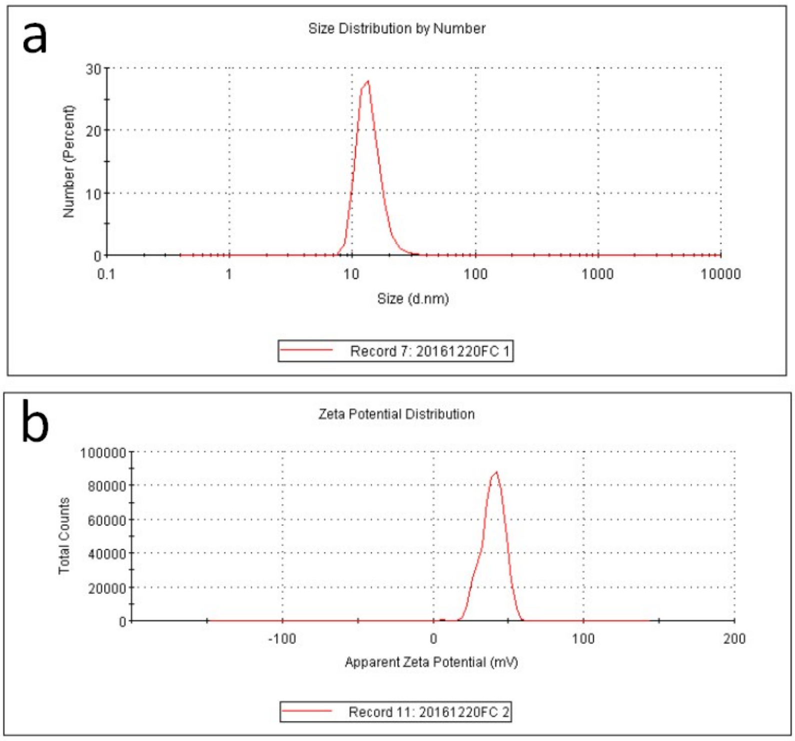

Figure S1. Size and Zeta potential of FCRAN. The average size(a) and zeta potential(b) is 13.76±0.2 nm and 24.6±0.1mv，respectively.

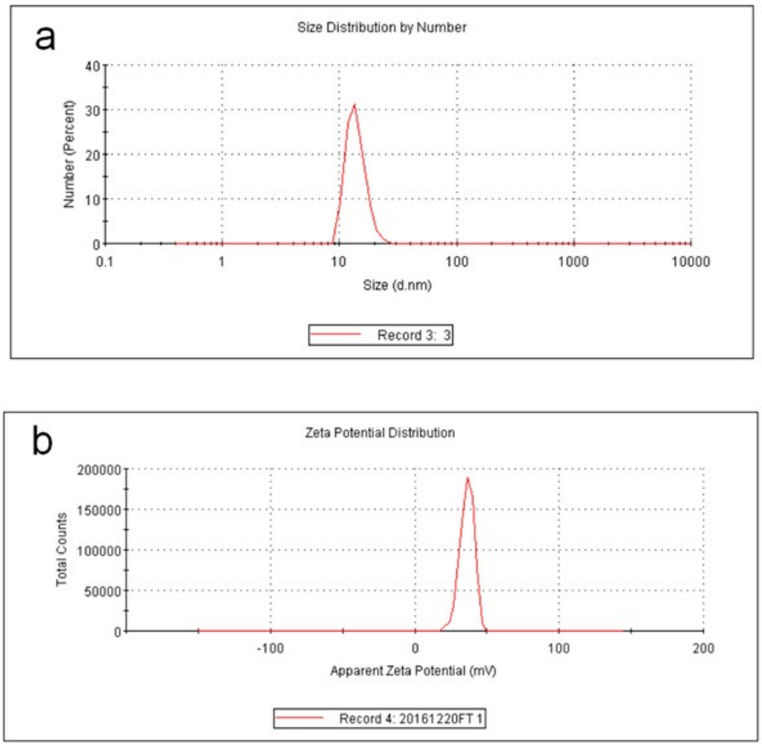

Figure S2 Size and Zeta potential of FCRAN/T. The average size(a) and zeta potential(b) is 13.86 ±0.1nm and 22.8±0.3 mv，respectively.

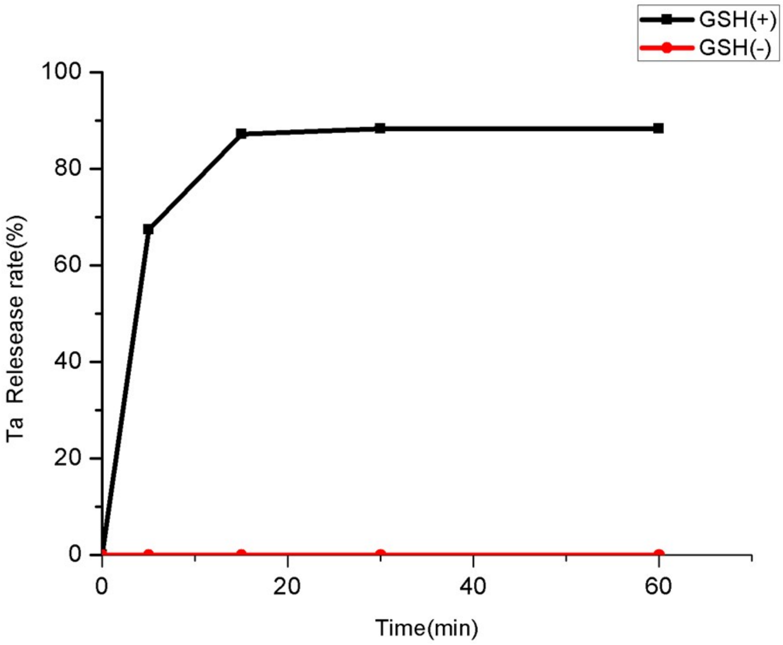

Figure S3. Release ratio of Taxol (Ta) at different time with/without GSH. there was no drug release during the test period in the absence of GSH; FCRAN/T dissociated rapidly in the presence of GSH, about 70% of the drug was released within 5 minutes, and the drug release reached the maximum at 15 minutes, about 90%. No further release of the drug was observed, possibly due to the total consumption of GSH.

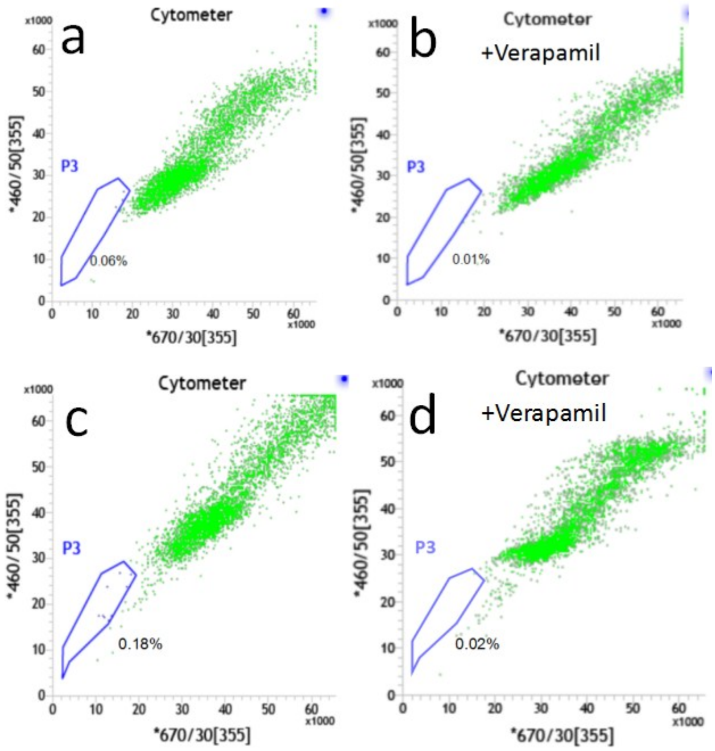

Figure S4. Identification of the cancer stem cells (side population cells, shown by SP cells) in human cancer cell lines. CAL-27(a,b) and KB (c,d) cells were stained with Hoechst33342 together with or without verapamil, analyzed by BD influx flow cytometer. Images of a and c were obtained from the specimens stained with Hoechst 33342 only, and those of b and d were from the specimens stained Hoechst 33342 by co-treating with verapamil.

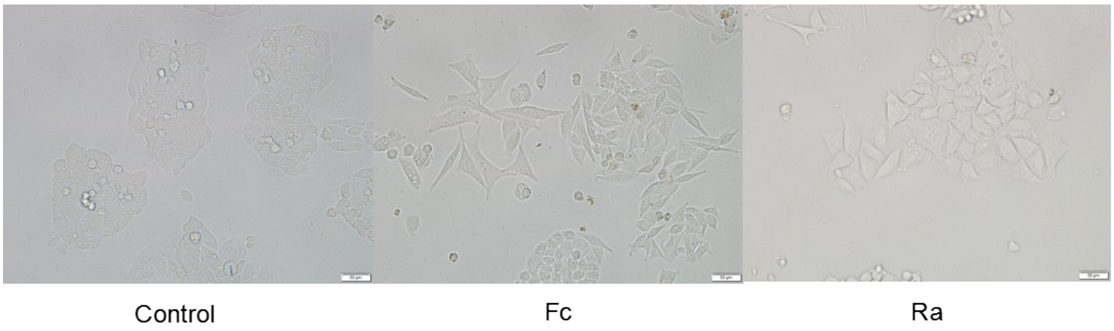

Figure S5. Microscopic images of the cancer stem cells in cell clones of each group. It could be seen that the cell clones of control group were more and growing better than FCRAN(Fc) and Ra group.
